# Supplementary material for: The Homocysteine and Metabolic Syndrome: A Mendelian Randomization Study
Source: Nutrients. 2021 Jul 16;13(7):2440. doi: 10.3390/nu13072440 (PMC8308555; doi:10.3390/nu13072440)
Supplement: Supplementary file 1 [file nutrients-13-02440-s001.zip › nutrients-1293997-supplementary.pdf]

Table S1. Summary of two independent Korean cohorts (HEXA and CAVAS) on MetS status.

Variables	HEXA			CAVAS		
	**CASE (N=676)**	**Control (n=3,017)**	**p-value**	**CASE (n=1,052)**	**Control (n=764)**	**p-value**
Female,%	45.6	57.6	<0.01	49.6	65.4	<0.01
Age, years	57(50,64)	51(46,58)	<0.01	62(55,65)	64(61,66)	0.92
BMI, kg/m2	26(24.5,27.8)	23.3(21.7,25.1)	<0.01	25.7(23.8,27.6)	22.9(21.1,24.7)	<0.01
WC, cm	91(86,95)	81(75,86)	<0.01	89.75(84,95)	82(77,87)	<0.01
FAG, mmol/L	98(89,116)	89(83,95)	<0.01	115.5(94,140)	92(86,104)	<0.01
TG, mmol/L	174(129,226)	92(65,128)	<0.01	179(132,247)	104(80,135)	<0.01
HDL-C, mmol/L	44(38,49)	55(48,64)	<0.01	40(35,46)	49(43,56)	<0.01
BP, mmHg						
Systolic	130(120.5,138.5)	120(110,130)	<0.01	138(126,150)	130(118,140)	<0.01
Diastolic	80(75,90)	78.5(70,80)	<0.01	87(80,90)	80(74,90)	<0.01
T2D, %	156 (23.08)	93(3.07)	<0.01	537(51.09)	119(15.58)	<0.01

Continuous variables were described as or median (interquartile range) and categorical variables as %. P value were calculated by Wilcoxon rank-sum test for continuous variables and chi-square test for categorical variables. BMI, body mass index; WC, waist circumference; FAG, fast glucose; TG, triglyceride; BP, blood pressure; T2D, type 2 diabetes.

Table S2. Summary of genotyping and quality control for outcome data.

Study	Genotype Array	Sample QC criteria	SNP QC criteria	Final Sample Size for Statistical Analysis
HEXA	Affymetrix Genome-Wide Human SNP array 6.0	more than one missing phenotype	SNP call rate <95%; HWE p-value < 10E-6	3,693 (case: 676, control: 3,017)
CAVAS	Affymetrix Genome-Wide Human SNP array 6.0	more than one missing phenotype	SNP call rate <95%; HWE p-value < 10E-6	1,814(case: 1,052, control: 764)

Table S3. The association between metabolic syndrome and potential confounders in KARE cohort.

Confounders	OR(95% CI)	p-value
Smoking	1.08 (0.97,1.2)	0.15
Drinking	0.96 (0.9,1.03)	0.32
RFS	0.99 (0.98,1.00)	0.30
PA	0.99 (0.99,1.00)	0.42
BMI	1.36 (1.32,1.39)	<0.01

All is adjusted by age, sex and area. RFS, recommended food score; PA, physical activity; BMI, body mass index.

Table S4. Instrumental variables associated with blood Hcy and MetS.

SNP	Chr	Position	beta.ex	se.ex	p-value	MAF
rs11121828	1	11757041	-0.036	0.006	4.74E-09	0.810
rs4845882	1	11765754	-0.039	0.008	2.74E-10	0.820
rs12404124	1	11796456	-0.037	0.006	1.35E-09	0.810
rs198391	1	11799004	-0.038	0.006	7.26E-10	0.810
rs2076001	1	11801854	-0.047	0.008	5.67E-09	0.900
rs12567136	1	11806318	-0.049	0.008	3.23E-10	0.900
rs2076003	1	11806734	-0.046	0.008	9.30E-09	0.900
rs7537765	1	11809890	-0.058	0.008	4.44E-13	0.900
rs535107	1	11812055	-0.037	0.006	2.63E-09	0.810
rs2236797	1	11815237	-0.046	0.008	1.12E-08	0.900
rs2075538	1	11819189	-0.048	0.010	7.86E-10	0.890
rs2075539	1	11820345	-0.046	0.008	7.23E-09	0.900
rs1023252	1	11821620	-0.039	0.009	8.03E-10	0.890
rs5063	1	11830235	-0.045	0.008	1.74E-08	0.900
rs1801131	1	11854476	-0.041	0.009	3.10E-11	0.830
rs1801133	1	11856378	0.073	0.005	5.53E-17	0.550
rs4846064	1	11857429	-0.040	0.005	5.53E-17	0.500
rs11588551	1	11864523	0.027	0.007	1.67E-08	0.560
rs6667637	1	11870544	-0.035	0.005	4.47E-13	0.580
rs2336377	1	11872557	0.037	0.007	8.07E-14	0.620
rs1321073	1	11874213	-0.029	0.007	2.04E-09	0.550
rs34175640	1	11874799	-0.029	0.007	7.84E-10	0.550
rs11800086	1	11894336	-0.035	0.005	7.35E-13	0.610
rs2336384	1	11968650	0.040	0.005	1.18E-16	0.580
rs1836860	3	187919298	-0.032	0.005	5.03E-09	0.730
rs1624230	3	187921629	-0.027	0.005	3.81E-08	0.650
rs1648700	3	187932610	-0.030	0.005	1.09E-08	0.720
rs1624569	3	187932763	-0.030	0.005	1.11E-08	0.720
rs1836883	11	88904815	0.029	0.005	9.55E-10	0.550

Table S5. The association between each instrumental variable and confounding factors.

Instrumental variable	Confounding factors (betas(standard error), p-value)			
	**Smoking**	**Drinking**	**RFS**	**BMI**
rs12567136	0.22(0.32),0.50	-0.11(0.29),0.70	1.21(1.03),0.24	-0.05(0.38),0.89
rs2336377	-0.06(0.12).0.60	0.03(0.09),0.72	0.10(0.31),0.75	0.11(0.12),0.35
rs1801133	0.02(0.11), 0.85	0.01(0.08),0.91	-0.54 (0.28), 0.05	-0.05(0.11),0.62
rs1624230	-0.15(0.13),0.25	0.12(0.10),0.22	0.12(0.34), 0.73	-0.19(0.13), 0.15
rs1836883	0.16(0.11),0.13	0.03(0.08),0.73	-0.40(0.27),0.15	-0.08(0.10),0.44

RFS, recommended food score; BMI, body mass index. smoking: current smoke vs non-, ex-smoker; drinking: current drinker vs non-, ex-drinker. Analyses was adjusted for age, sex and area. Genetic model is additive.

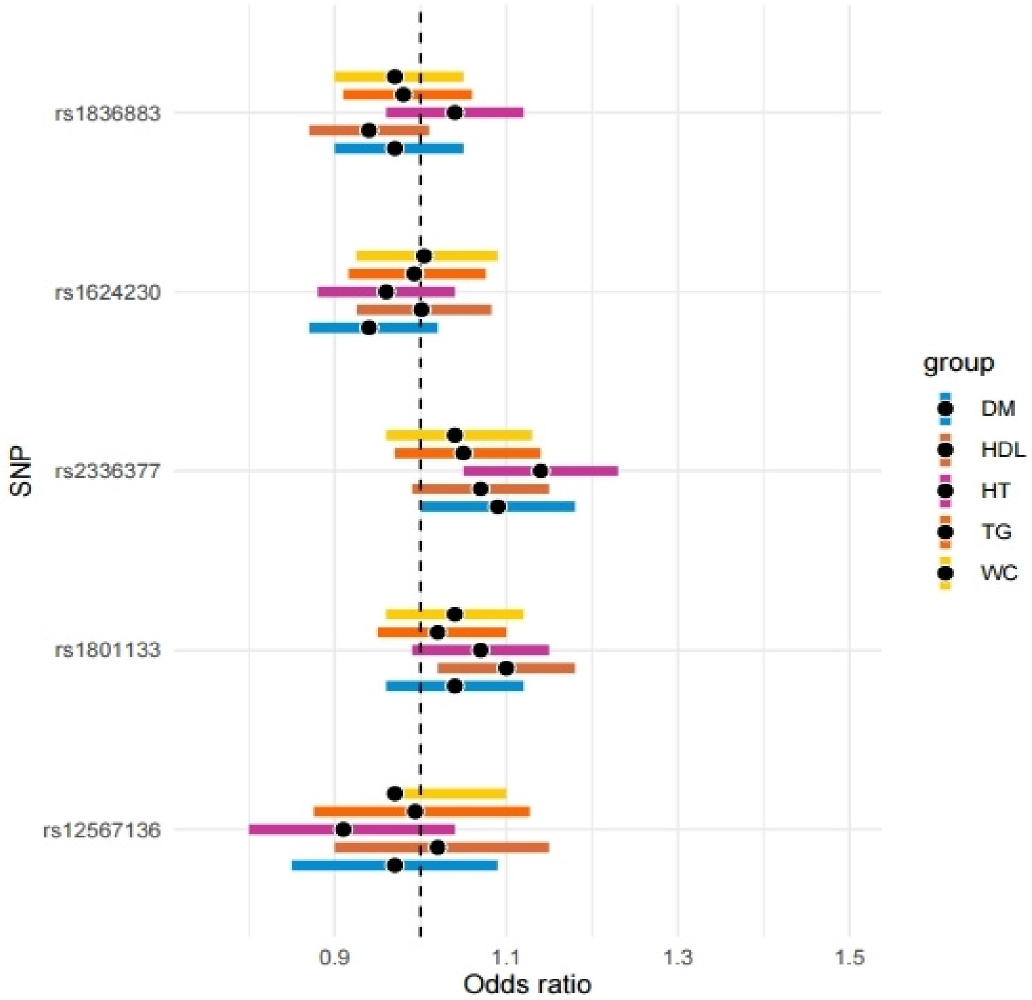

Figure S1. Odds ratio of five selected SNPs and individual components of MetS in KARE. DM, diabetes; HDL, high density lipoprotein; HT, hypertension; TG, triglyceride; WC, waist circumference.
